# Impact of Interactions with a Puppy and Handler Versus a Handler Alone on Stress and Vitality in a University Setting: A Crossover Study

**DOI:** 10.3390/ani14172454

**Published:** 2024-08-23

**Authors:** Tiffani J. Howell, Dac L. Mai, Pam Draganovic, John-Tyler Binfet, Pauleen C. Bennett

**Affiliations:** 1 School of Psychology and Public Health, La Trobe University, Bundoora VIC 3086 and Bendigo VIC 3552, Australia; jimmy.mai@latrobe.edu.au (D.L.M.); p.draganovic@latrobe.edu.au (P.D.); pauleen.bennett@latrobe.edu.au (P.C.B.); 2 Faculty of Education, University of British Columbia, Kelowna, BC V1V 1V7, Canada; johntyler.binfet@ubc.ca

**Keywords:** human–animal interaction, university student wellbeing, stress reduction, therapy dog, visitation dog

## Abstract

**Simple Summary:**

Campus-based dog programs can reduce stress and enhance wellbeing in university students and staff. However, most research has focused on adult dogs, who are well-trained and accompanied by a handler. In our study, participants came to the university campus for 20 min interactions with an assistance dog puppy in training and their handler during one visit, and the handler alone in another visit. We asked participants about their stress and vitality levels before and after each interaction. Stress was lower after the interaction with the puppy and handler compared to the handler alone. Vitality was greater after both visits among participants who had their session with the puppy and handler first. This study indicates that assistance dog puppies in training, with knowledgeable handlers, can be effective in campus-based visitation dog programs.

**Abstract:**

Brief interactions with a well-behaved dog can enhance wellbeing, but most campus-based dog visitation programs employ adult, trained dogs. There is little research about the impact of puppies on wellbeing or stress in this context. The aim of this study was to examine changes in perceived stress and vitality after interacting with a puppy. Staff and students (*N* = 32) at an Australian university attended the campus on two occasions, one week apart, as part of a crossover design. Participants were pseudo-randomly allocated to a group whereby they spent 20 min interacting with a handler alone at Visit 1 and interacted with a puppy and handler at Visit 2, or another group which reversed the interaction order. Perceived stress and subjective vitality were measured before and after each interaction. The increase in vitality was greater in the group experiencing the puppy and handler interaction first (significant main effect, *F*(1,49) = 646.89, *p* = 0.024, η^2^_p_ = 1.00), regardless of the visit, possibly due to a social lubricating effect by the puppy, which carried over to the ‘handler alone’ second visit. Reductions in perceived stress were greater after the interaction with the puppy, for both groups (significant interaction effect, *F*(1,49) = 5.13, *p* = 0.029, η^2^_p_ = 0.11), indicating that the puppy’s presence can reduce stress more than the handler alone. This extends the evidence for university-based dog-facilitated wellbeing programs, by showing that interactions with puppies can also be effective. This is important as it may mean that puppies already on campus as part of a socialization/training program can be incorporated into wellbeing programs for staff and students.

## 1. Introduction

University students are at risk of poor mental health outcomes [1], especially international [2] and gender diverse [3] students. This can result in compromised learning and possible attrition from their degree program [4]. Thus, universities have a vested interest in optimizing the mental health of students, as this holds potential to engage students in their studies, reduce attrition, and support students in the completion of their post-secondary degree. Despite attempts by universities to improve outcomes for students, there is still work to be done, as nearly 87% of students felt that their studies and wellbeing were negatively impacted by COVID-19 [5], students felt less emotionally engaged in online learning contexts compared to traditional learning environments [6], and mental health among university students remains worse than before the pandemic onset [7].

Staff at universities also face many challenges and many experienced mass redundancies and non-renewal of contracts during COVID-19. The staff who remain continue to experience high levels of work-related stress [8] as change processes upset the status quo and staff are required to fill work voids left by others [8]. 

It is clearly important to provide both students and staff with ways to reduce stress levels. There is evidence that positive interactions with dogs can increase feelings of wellbeing and reduce stress [9] and homesickness [10]. Dogs are reported to offer non-judgmental support, whereas human support may be perceived as judgmental [11]. Existing visitation dog programs at university campuses often employ volunteer teams comprised of a trained adult dog and their handler [12]. Due to the assessment procedure required by some programs [12], which can likely only be met by adult dogs, puppies are not typically integrated into these programs. Nonetheless, there is evidence that people prefer to interact with puppies compared to adult dogs [13], making it possible that puppies, even if not fully trained, would also be able to help reduce stress and enhance feelings of wellbeing. Since people may prefer to interact with puppies, it is possible that they would also be able to help reduce stress and enhance feelings of wellbeing.

Wellbeing has been conceptualized in different ways with various elements [14]. A sense of vitality, which refers to feeling alive and energetic, is part of wellbeing, and it has been used as both a trait and state measure. Therefore, vitality could be a useful proxy for wellbeing in short timescales, like brief interactions with puppies.

A Dogs on Campus program is offered at the two largest campuses of La Trobe University (LTU) in the Australian state of Victoria: Bundoora, in greater Melbourne, and Bendigo, in regional Victoria. In this program, students and staff act as puppy raisers for assistance dog puppies in training [15,16], between the ages of 12 weeks and approximately 14 months. The puppies live with the puppy raisers and accompany them to campus each day, and the puppy raisers receive weekly dog training on campus by staff employed by our assistance dog training provider partner, Dogs for Life. As part of an ongoing investment in evidence-based program development, the program provider incorporated feedback from puppy raisers in recent studies [15,16], indicating that they would benefit from being able to leave their puppy at the on-campus training facility during their workday, if required. This gives the raisers some respite from the responsibilities of 24/7 puppy care, and enables the puppies to receive additional training from Dogs for Life staff. It also means that puppies and young dogs are available to engage in other, supplementary activities.

Previous research suggests that, beyond being involved in a formal puppy raising program, the wider campus community, including students outside the puppy raising program, could benefit from safe, positive interactions with dogs [17]. For this reason, we established a program at LTU whereby staff and students can spend time with a Dogs for Life assistance dog puppy-in-training in a safe, relaxing way. Called Puppies Assisting Wellbeing for Students and Staff (PAWSS), this program was based on an existing program at University of British Columbia in Canada called Building Academic Retention through K-9s (B.A.R.K.). B.A.R.K. employs visitation dogs [18] who have been temperamentally assessed as suitable for visiting work (i.e., are calm and comfortable among unfamiliar people), and who have undergone behavior training with their owner/handler to engage in volunteer visitation programs [9,10]. Unlike the B.A.R.K. dogs, who are fully grown, calm, and relaxed, the puppies in the PAWSS program at LTU are still learning various skills and engaging in socialization with skillful Dogs for Life staff. Although they are carefully screened for suitability before selection into the program, unlike the fully trained and calm dogs in the B.A.R.K. program, the PAWSS puppies can be either playful or calm during interactions. Nonetheless, it is likely that a positive interaction with a puppy, even if not especially relaxing, may be beneficial to the wellbeing of students and staff who like dogs. Most research has focused on students [9,10], because reduced stress may enhance educational outcomes. Nonetheless, with the reported levels of work-related stress in university staff [8], this population would probably also benefit from participating in a stress-reduction program. It is reasonable to expect that the lower levels of stress among university staff may translate to better support services, and potentially improved educational outcomes for students. 

The aim of this study was to investigate the effects of a positive, short-term interaction with a puppy and handler, versus a hander alone, on perceived stress and vitality, as proxies for subjective wellbeing. We hypothesized that perceived stress and vitality would improve more after an interaction with a puppy and handler, compared to the handler alone, but that there would be some degree of improvement in both conditions due to having a positive social interaction with the handler.

## 2. Materials and Methods

This project received approval from both the LTU Human (approval number: HEC22163) and Animal (approval number: AEC22017) Ethics Committees. 

### 2.1. Participants

Participants were recruited using university internal communications and social media, as well as by posting flyers around the Bundoora campus. The recruitment ads indicated that there would be an interaction with a puppy and handler on one visit and the handler alone on another visit. Participants contacted the research team by email to express interest in the study and to confirm eligibility to participate (i.e., 18+ years old, staff or student at the university, able to visit the Bundoora campus on two occasions about a week apart, and not afraid of or allergic to dogs). At that point, we booked their first visit to the DogHub, a lab and office space at LTU’s Bundoora campus. Half (approx. 30 m^2^) of the 60 m^2^ office space was used for this study. Data collection proceeded over four months between September and December 2022.

University Students and Staff: The participants were 22 students, 22 staff members, and 1 student who was also a staff member (*N* = 45) from the LTU Bundoora campus, of whom 32 participants attended both sessions required for participation. While we collected data on each participant’s affiliation with LTU, this was not expected to influence their study outcomes, so all participant groups were combined for analysis. Among the participants who attended both sessions, nearly all (*n* = 30) were women, and 20 were from Australia. Another 4 were from the United Kingdom, and 1 each was from Vietnam, Spain, South Korea, the United Arab Emirates, Sri Lanka, Singapore, China, and Mexico. Half of participants (*n* = 16) were staff members, while 12 participants were undergraduate students, and 4 were postgraduate students. Participant age ranged from 18 to 67 years (*M* = 34 years; *SD* = 12 years). Of the 13 participants who were unable to attend the second visit, 7 would have received the ‘handler alone’ condition in the second visit, and the remaining five would have had the interaction with the puppy and handler in the second visit.

#### 2.1.1. Canine Participants

There were nine puppies in this study, all of whom were assistance dogs in training with Dogs for Life, our assistance dog training partner. There were four Golden Retrievers, three Labrador Retrievers, one Australian Shepherd, and one Lagotto Romagnolo. Five puppies were female and four were male. They ranged from 4 months old to 1 year and 4 months old (*M* = 10.5 months; *SD* = 3 months) at the beginning of data collection in September 2022. Canine welfare was not measured but was monitored via casual observations by two handlers from Dogs for Life, and there were no reports of compromised welfare over the course of the study. 

#### 2.1.2. Handlers 

The two handlers were Dogs for Life staff members responsible for training the puppies. Both handlers were female; this was not a planned part of the study, but happened because the Dogs for Life staff members who regularly attend LTU as part of their puppy training program both happen to be female.

### 2.2. Measures

Demographic information (i.e., age, gender, country of birth, staff or student, and length of time in Australia if born overseas) was collected as part of the Visit 1 pre-interaction survey. The survey included a single-item visual analogue scale asking participants to ‘please rate your stress level right now,’ with response options ranging from 1 = *not stressed at all* to 5 = *very stressed*, with 3 = *neutral*. This measure has been employed in previous research on the B.A.R.K. program [9,19]. While single-item scales are not common, they are acceptable when used to measure a global construct like stress [20,21,22], with a good construct validity reported by Elo et al. [20]. Nonetheless, survey measures of stress are not objective, physiological measures. Participants also completed the Subjective Vitality Scale [SVS; 23], as a proxy for wellbeing. It is a 7-item scale which asks participants to rate the extent to which the relevant statement (e.g., ‘*At this moment, I feel alive and vital*’) is currently true for them. Items are scored on a 7-point Likert scale ranging from 1 = *not true at all* to 7 = *very true*. The SVS was selected because it has been validated for use in short timescales, has a good construct validity, and had an alpha level of over 0.80 in the original study [23].

### 2.3. Procedure

**Figure 1:**
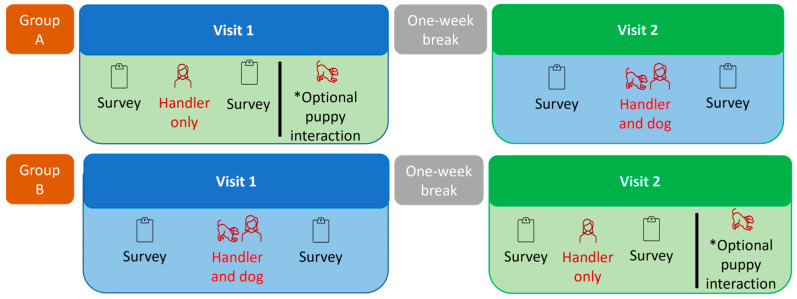
Flow chart of the crossover research design for this study. Participants had both an interaction with a dog and handler, and an interaction with the handler alone, but half had the interaction with the handler alone first (i.e., Group A), and the other half had the interaction with the dog and handler first (Group B). * After the ‘handler alone’ session and survey completion, both groups were invited to have an interaction with a puppy before leaving the DogHub.

This project employed a crossover design, whereby each participant participated in two sessions. For half of the participants (i.e., Group A), the first session included a 20 min interaction with a handler alone, and the second visit included an interaction with a puppy and handler. For the other half of participants (i.e., Group B), the sessions were reversed, with the interaction with a puppy and handler occurring first (see Figure 1).

Applied research of this nature is complicated to conduct as it involves multiple parties (i.e., students, volunteer handlers, puppies, university staff, etc.) and data collection in a busy context. We ensured implementation fidelity by ending all interactions after 20 min and tracking participation. We did not monitor the interaction in real time, however, because the ability to speak privately with the handler was an important component of the study. We wanted to create a relatively naturalistic atmosphere in which the conversation could flow as organically as possible, and monitoring the interaction (e.g., via video link) may have impeded this goal. Participants could choose to sit in a chair next to the handler, or on a large bean bag cushion on the floor to be close to the puppy; based on their location at the end of the session, most participants appeared to prefer the chair. Handlers were free to share information about their puppy and engage visitors in discussion as they saw fit. For the same reason, the interactions were not video- or audio-recorded for later analysis.

The handlers were not given a script for how to engage with participants during the 20 min interaction. This was because our study involved individual participants spending time with the handler/puppy, rather than most other research, in which a handler/dog would interact with a few participants at a time [10]. Because of the potential dynamics inherent in group discussions, a script is advisable. With one-on-one interactions, we believed the interaction would be more authentic if the conversation flowed freely, and we wanted participants to be able to lead the conversation if they desired. To get the conversation started, handlers typically asked questions about the participant’s studies, why they chose the university for work or study, and whether they had any pets at home. The handlers then allowed the conversation to be led by the participant, and a few participants preferred to speak mainly about dogs, even in the absence of the puppy. However, for most participants, the handlers indicated that the conversation covered a variety of topics.

Prior to the study, the handlers received instructions on how to respond to self-harm disclosures by the study participants, should they arise. This included notifying the lead author, who would immediately contact the University Counselling Service and ask them to follow up with the participant, with the participant’s full knowledge. No such disclosures were made.

#### 2.3.1. Visit 1

When participants arrived for their first visit, they completed the consent form and then completed a short survey. The first questions were demographic, and the remaining questions were the single-item perceived stress measure and the SVS. After completing the survey, the handler entered the room either alone, or with a puppy, depending on the condition. The research team left the area so that the handler and participant could speak privately and without interruption (i.e., the interaction). After 20 min, the research team re-entered the room and the handler left, with the puppy, if applicable. The participant then completed the post-session survey. The post-session survey was identical to the measure used in the pre-test session, except that the demographic questions were not included and there was an optional comment box at the end. The participant was offered a snack as a ‘thank you’ for participating, their second visit was booked for one week later, and they left the DogHub. Participants who had the interaction with the handler alone for the first visit were offered the chance to meet some of the puppies in the next room at this point, to avoid disappointment at not having been able to spend time with a puppy.

#### 2.3.2. Visit 2—One Week Later

The following week, participants returned to the DogHub for their second session. The second session proceeded identically to the first session, except that the demographic questions were not asked on the survey. The condition was also interchanged, whereby if the participant had interacted with the handler alone in Visit 1 (i.e., Group A), the handler brought a puppy with them for Visit 2, and vice versa.

Whenever possible, the same handler was present for both interactions. In the case of six participants, this was not possible. Four of these participants received the handler alone in Visit 1, and the remaining two participants received the puppy and handler in Visit 1. For interactions with a puppy, the handler selected the puppy that she deemed most appropriate for the interaction at that time. This was determined partly by puppy availability, as not all puppies were available on all data collection dates, as well as the handler’s informal assessment of whether the puppy risked being overstimulated by the interaction, given their young age.

### 2.4. Analysis

The crossover design in this study allowed each participant to serve as their own control. The analyses were conducted for perceived stress and vitality.

All analyses were conducted using SPSS Statistics, a statistical package software for social research (IBM, Armonk, New York, USA), and JAMOVI v.2.3.28.0, an open-source statistical software package [24]. A linear mixed-effect model analysis was used to test the hypotheses considering the crossover design of this study. We opted for a linear mixed-effect modeling as an alternative to the more conventional two-way mixed-model (split-plot) ANOVA because it is preferable in repeated measures designs [25], and it can optimize the balance between Type I errors and power in small sample sizes [26]. Assumptions of the mixed-effect model were checked for each outcome measure separately. The normality of residuals and homogeneity of variance were tested using Shapiro–Wilk’s and Levene’s tests, respectively. The assumption of normality of residuals was only violated for perceived stress at Visit 1, while it was upheld for all other variables. Linear mixed-effect models are robust to violations of normality since this violation does not result in bias estimates [27]. The assumption of homogeneity of variance was upheld for all outcome measures.

## 3. Results

### 3.1. Descriptive Statistics

Mann–Whitney U-tests showed that there were no differences between the groups based on demographic factors, including gender, age, country of birth, time in Australia, and type of affiliation with the university (all *p* > 0.252). Wellbeing measures were obtained for each group at each visit, resulting in four data points for each participant: before and after the interaction in the first visit, and before and after the interaction in the second visit. Then, the difference between the pre- and post-interaction measures was calculated for each visit. Table 1 provides descriptive statistics of the wellbeing measures for each group by visit, and the total group measures from both visits.

### 3.2. Linear Mixed-Effect Model

#### Main Effects of Group and Visit on Wellbeing Measures

Table 2 provides details of the mixed-effect model results for the main effects of the group and the visit, and the interaction effect for both outcome measures. The independent variables and interaction terms were included in the same model for each outcome measure to account for the influence of one another. For instance, the effect of the group (fixed effect) on vitality was calculated when accounting for the random effect of the visit and the interaction between them. At the same time, the random effect of the visit was calculated when accounting for the effect of the group and the interaction on vitality.

As can be seen in Table 2, the main effect of the group was significant for vitality, *F*(1,49) = 646.89, *p* = 0.025, η^2^_p_ = 0.99. In other words, regardless of the visit, participants who were allocated to Group B (puppy and handler first) saw a significantly greater increase in vitality compared to Group A participants (handler only first), with a large effect size. No significant main effects for the group were observed for perceived stress.

No significant result was found for the random effect of the visit. This means that after accounting for the influence of the activities, the differences in wellbeing measures at Visit 1 did not differ significantly from Visit 2.

**Figure 2:**
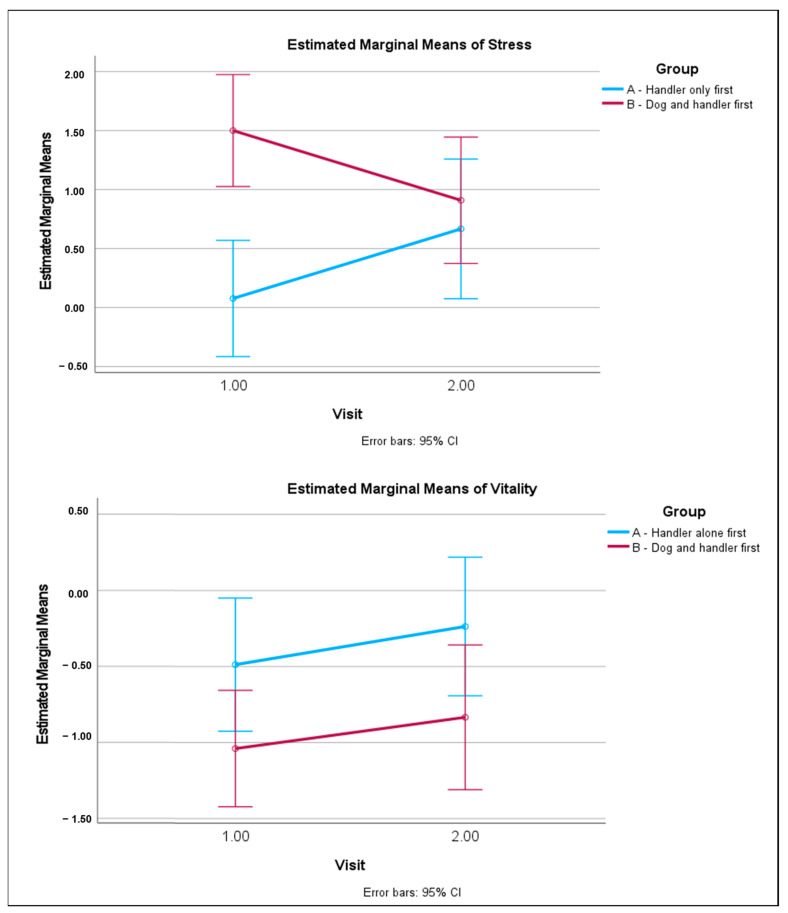
Estimated marginal means of stress and vitality between groups at different visits. Changes between pre- and post-interaction scores are reported, in which a higher score means a larger reduction after the interaction.

The interaction effect between the group and the visit was significant for perceived stress, *F*(1,49) = 5.13, *p* = 0.029, η^2^_p_ = 0.107, with a large effect size. This means that, for perceived stress, how participants of different groups rated their perceived stress was dependent on whether they were at Visit 1 or Visit 2. The visual representation of this significant interaction is shown in Figure 2. No significant interaction effects were found for vitality, also shown in Figure 2.

As can be seen in Figure 2, participants in Group B experienced a much larger reduction, with a large effect size, in perceived stress at Visit 1 (puppy and handler present) than did participants in Group A (handler only present). When swapping activities at Visit 2, the reduction in the perceived stress of Group B (handler only present) was reduced compared to Visit 1, while Group A participants experienced a greater reduction in perceived stress during Visit 2 (puppy and handler present) than they did during Visit 1. Thus, within each group, participants experienced a greater reduction in perceived stress after the interaction with the puppy and handler compared to after the interaction with the handler alone.

To examine the simple main effects, we compared the mean differences between Visit 1 and Visit 2 for Group A and Group B separately, for both outcome measures. There were no significant differences for either outcome measure. We also compared the mean differences between Group A and Group B at each visit for both outcome measures (Table 3).

The simple main effects analysis revealed that, for perceived stress, there were significant mean differences between Group A (handler alone first) and Group B (puppy and handler first) at Visit 1, indicating that the interaction with a puppy led to a greater reduction in perceived stress compared to interacting with humans. However, at Visit 2, there were no significant differences between the groups.

## 4. Discussion

This study used a crossover design to measure participants’ perceived stress and sense of vitality before and after a 20 min interaction with a puppy and handler, or with the handler alone. Our hypotheses were partially supported. Statistically significant changes between pre and post scores were observed for vitality, with participants in the ‘puppy and handler first’ group experiencing a greater increase in vitality than participants in the ‘handler alone first’ group, regardless of the visit (see Table 2). In addition, we found an interaction effect for perceived stress, with participants in both groups experiencing a greater reduction in perceived stress in the puppy and handler condition than in the handler only condition, regardless of whether they experienced this in the first or second visit. This was particularly pronounced in the puppy and handler first group (see Table 3 and Figure 2). At Visit 1, participants who interacted with the puppy and handler experienced a very large reduction in perceived stress, while those in the handler alone condition showed no measurable pre post change. At Visit 2, both groups showed a similar reduction in perceived stress after the interaction. For Group A, where Visit 2 involved interacting with a puppy and handler, this change was larger than seen in Visit 1 (the handler only condition). For Group B, where Visit 2 involved interacting with a handler only, the change was considerably smaller than observed in Visit 1, where the handler was accompanied by a puppy. This was as we hypothesized: that positive interactions with another human would likely have some benefit for participants, but that interaction with a handler and puppy together would result in larger reductions in stress. The improvement in stress levels accords with previous research investigating similar campus-based programs with dogs [9,10,28]. Social support can have a buffering effect on stress [29], which is likely why a small stress reduction was observed in the handler-alone condition for both groups.

There was no main effect of the group on perceived stress, which we expected because we anticipated that the presence of the puppy would have a stronger influence on stress than whether the puppy was part of the first or second visit. That is, we had anticipated an interaction effect of the group by visit, which we observed. However, there was a main effect of the group on vitality. The effect on vitality may be due to a novelty effect of meeting a puppy in the first visit. There is evidence that novelty alone can enhance wellbeing [30]. Alternatively, there may have been a social lubrication effect of having the puppy present in the first visit that enhanced participants’ sense of vitality, which then carried over to the second visit without the puppy.

There have been several studies exploring university-based dog interaction programs, many of which observe wellbeing improvements and reductions in perceived stress among participants after the interaction, e.g., [9,10,28]. A systematic review and meta-analysis of visitation programs in higher education found improvements in mental health (e.g., stress and anxiety) but no improvements in physiological health or cognitive functioning [31]. The mechanisms of change from dog programs at universities are still unknown, but there is evidence that the ability to directly touch the dog is more effective in reducing stress than merely being near the dog [19,32]. In our study, when participants interacted with the puppy and handler, there were no explicit restrictions on how the interaction proceeded, provided that the handler was comfortable, and that the puppy’s welfare was ensured. Following up the impact of direct physical interaction is recommended for future studies.

A novel aspect of our study was the inclusion of puppies in the interaction, rather than fully grown, temperament-assessed visitation dogs. Most campus-based wellbeing programs with dogs (e.g., B.A.R.K) work with volunteer handlers who bring in calm, friendly dogs that have been assessed as suitable for this type of work [9]. This approach helps to ensure safety for all parties, as selected dogs are low-risk for behaviors like biting people, and they are known to enjoy positive interactions with unfamiliar people, thus protecting their own welfare. To our knowledge, our study was the first to incorporate puppies into the program. Because our Dogs on Campus program is fully supported by the university’s administrators, a group of assistance dog puppies in training are typically on campus during weekdays. These puppies are selected specifically for temperament and physical health while still in the litter, and then extensively socialized and trained, to prepare them for assistance work as adult dogs. Socialization is important for all puppies [33,34], and especially so for assistance dogs in training, who will be expected to encounter a large variety of different people, places, and objects over their working lives [35].

Many puppies would not be suitable for campus wellbeing programs due to undesirable temperament traits or anxiety/disinterest when interacting with unfamiliar people. Assistance dog puppies in training are likely to be suitable for these interactions due to their selection for a calm, robust temperament. They may also benefit from these interactions due to the socialization experiences inherent in campus-based wellbeing programs. They will have the opportunity to meet lots of different types of people in a controlled environment with a skilled handler who will prioritize their welfare. Indeed, a criticism of animal-assisted services (e.g., animal-assisted education, animal-assisted treatment; [36]) in general has been an emphasis on the benefits for the people involved, with less focus on the impacts on the animal or whether the animal is consenting to be involved in the process [37]. There have therefore been calls to involve animals in programs designed to benefit both the people participating and the animals themselves, such as in rehabilitation programs with shelter dogs [37]. According to our results, while involving puppies in campus-based wellbeing programs may not be feasible for some puppies and should only be done with the support of highly knowledgeable dog handlers, it could represent a mutually beneficial program in the right circumstances. The use of technologies like social media to disseminate research findings can be appealing to young people [38] and may be a good way to inform the wider community of these findings.

Unlike other studies, e.g., [9,10,39], in which each dog and handler team engages with a small group of participants, in this study, the interactions proceeded with just one participant at a time. This may have provided each participant with more freedom to interact with the puppy in the way they preferred, rather than possibly feeling constrained by the presence of other group members. Future research may employ video recording and behavioral coding to measure the ways in which participants and dog-handler teams interact within sessions to elucidate the mechanisms contributing to optimal wellbeing outcomes. For instance, it is possible that the puppy sees the handler as a safe haven [40], and this may impact the handler’s behavior with, compared to without, the puppy present. It is also possible that the participants who did not get to visit the dog in the first visit were disappointed by this outcome, which may have temporarily increased their stress levels when completing the post-session survey, which was before they were permitted to meet a puppy. Building from this, a future study comparing one-on-one interactions versus group-based interactions would help to determine whether socialization is an important dimension of canine-assisted stress-reduction interventions.

### Strengths, Limitations, and Future Directions

The current study is one of the few investigations, to date, of university visitation dog programs using a crossover design. A previous study used a crossover design comparing a 15 min interaction with a dog and handler and a non-social control condition [41]. These researchers measured perceived stress and alpha amylase, a measure of physiological arousal. As in our study, they found a decrease in perceived stress when interacting with the dog and handler compared to the control condition, with a large effect size. Barker et al. [41], showed no change in alpha amylase. More research on this topic using physiological measures is needed.

This study employed a small sample size with mostly women and, as such, the results should be interpreted with that in mind. This is important for this study because women experience a greater oxytocin increase than men after interacting with their pet dog when they arrive home from work [42]. Since oxytocin can reduce stress [43], it is possible that the results we found would be less pronounced in a largely male sample. The use of a crossover design allows each participant to act as their own control and increases the power [44]. However, crossover designs are associated with a higher level of attrition than one-off data collection methods [44], and the attrition rate for the current study was nearly 30%. Unfortunately, we were not able to always work with the same handler for every participant, which introduced a potential confounding factor. Nonetheless, this method should be replicated in future studies to determine whether these results are generalizable beyond the current sample, ideally with one handler for all participants. Given the busy schedule of students and staff, future research may employ a longitudinal design with regular drop-in sessions where staff and students may come back at their preferred time to reduce the attrition rate. In addition to measures of wellbeing, data on the frequency and interval of their visit along with the duration and nature of interactions would be necessary. Additionally, while the handler was always present with the puppy to ensure that there were no risks to the puppy’s welfare, more research into animal welfare during these types of interactions is needed, as well as a comparison of adult dogs and puppies working in these roles. Finally, no script was provided for the handlers, so the nature of the conversations may have varied between participants; future research should control for this by providing a script, as was performed in Binfet et al. [19].

## 5. Conclusions

This study used a crossover design to measure perceived stress and vitality comparing a 20 min interaction with a puppy and handler versus a 20 min interaction with the handler alone. We found improvements in perceived stress after the interaction with the puppy and handler, regardless of whether this happened on the first or second visit, and an increased sense of vitality in the group that met with the puppy and handler on the first visit, even in the ‘handler alone‘ second visit. This study extends our understanding of the potential benefits of positive, short-term interactions with a dog within the post-secondary context. Also, it provides, to our knowledge, the first study of its type in which puppies were employed instead of specially trained adult dogs. Puppies training to be future assistance or therapy dogs must receive extensive socialization, so this program may have benefits for both human and canine participants. Future research should consider the welfare of dogs participating in stress-reduction initiatives and possible mechanisms explaining the benefits observed by participants in campus-based dog programs.

## Figures and Tables

**Table 1:** Descriptive statistics of wellbeing outcome measures for the group and the visit. Group A = Handler alone first. Group B = Puppy and handler first. Changes over time were calculated by subtracting the post-interaction score from the pre-interaction score. Therefore, a negative value indicates an increase in the variable after the interaction. For instance, vitality increased, while perceived stress decreased for both groups, in both visits.

Group	Visit	Vitality		Perceived Stress	
		*M* (*SD*)	*n*	*M* (*SD*)	*n*
A (Handler alone first)	1	−0.49 (0.89)	13	0.08 (0.64)	13
	2	−0.24 (0.61)	12	0.67 (0.71)	9
Total Group A		−0.37 (0.76)	25	0.32 (0.72)	22
B (Puppy and handler first)	1	−1.04 (0.77)	17	1.5 (1.22)	14
	2	−0.83 (0.85)	11	0.91 (0.7)	11
Total Group B		−0.96 (0.79)	28	1.24 (1.05)	25

**Table 2:** Summary of mixed-effects model results. The group was the fixed effect, while the visit was the random effect. Significant results are highlighted in gray.

Outcome Measure	Source	Sum of Squares	*df*	Mean Square	*F*	Sig.	η^2^_p_
Vitality	Intercept	21.74	1	21.74	32.27	0.111	0.97
	Error	0.67	1	0.67 ^a^			
	Group	4.26	1	4.26	646.89	0.025 *	1.00
	Error	0.01	1	0.07 ^b^			
	Visit	0.67	1	0.67	102.27	0.063	0.99
	Error	0.01	1	0.07 ^b^			
	Sequence * Visit	0.01	1	0.01	0.01	0.918	0.00
	Error	30.25	49	0.62 ^c^			
Perceived Stress	Intercept	28.37	1	28.37	7,317,025	<0.001	1.00
	Error	<0.001	1	3.88			
	Group	7.92	1	7.92	1.99	0.393	0.67
	Error	3.98	1	3.98 ^b^			
	Visit	<0.001	1	<0.001	<0.001	0.999	0.00
	Error	3.98	1	3.98 ^b^			
	Sequence * Visit	3.98	1	3.98	5.13	0.029 *	0.11
	Error	33.33	43	0.78 ^c^			

* Significant at 0.05; ^a^. MS (Visit); ^b^. MS (Sequence * Visit); ^c^. MS (Error).

**Table 3:** Mean differences of Group by Visit. Significant result is highlighted in gray. Group A = Handler alone first; Group B = Puppy and handler first.

Outcome Measure	Visit	(I) Group	(J) Group	Mean Difference (I−J)	SE	Sig	95% CI	η^2^p
Vitality	1	A	B	0.55	0.29	0.062	[−0.03; 1.13]	0.069
	2	A	B	0.60	0.33	0.075	[−0.06; 1.26]	0.063
Perceived Stress	1	A	B	−1.423 **	0.34	<0.001	[−2.11; −0.74]	0.291
	2	A	B	−0.24	0.40	0.543	[−1.04; 0.56]	0.009

** Significant at 0.001.

## Data Availability

To access the anonymized data file, please contact the corresponding author.
